# Supplementary material for: Neuroanatomical and behavioral characterization of corticotropin-releasing factor-expressing lateral Habenula neurons in mice
Source: Commun Biol. 2026 Jun 16;9:1280. doi: 10.1038/s42003-026-10490-8 (PMC13631236; doi:10.1038/s42003-026-10490-8)
Supplement: Supplementary file 1 [file 42003_2026_10490_MOESM1_ESM.pdf]

nature portfolio

Peer Review File

Neuroanatomical and behavioral characterization of corticotropin releasing factor-expressing lateral Habenula neurons in mice

Corresponding Author: Dr Fereshteh Nugent

This file contains all reviewer reports in order by version, followed by all author rebuttals in order by version.

Version 0:

Reviewer comments:

Reviewer #1

(Remarks to the Author)
In this ms, Flerlage, Gouty, Thomas and colleagues identify for the first time a population of CRF-expressing neurons in the lateral habenula (LHb). Using anatomical tracing, viral approaches, and CRH-Cre mouse lines (CRH-Cre and CRH-Cre:Ai14), the authors describe the input–output architecture of this CRF system in the LHb. They further employ a "gain-of-function" strategy, expressing excitatory DREADDs in LHbCRF neurons to investigate their potential roles in various behaviors, including locomotion, anxiety, reward processing, and innate fear. Notably, they observe altered behavioral strategies in the looming test (increased action-locking) and a sex-dependent difference in escape latency following agonist injection.
This work has the merit of highlighting a previously overlooked LHb neuronal population, and the inclusion of both sexes is a valuable strength. However, the study feels preliminary in several key aspects, and some conclusions are not sufficiently supported by the data. For instance, the first four figures lack quantification and rely only on representative images— a limitation when characterizing a novel neuronal population. Furthermore, the rationale for using a gain-of-function approach over a loss-of-function is not clearly explained, particularly given the causal interpretations drawn from the DREADD experiments. A functional description of these neurons under physiological conditions is also missing.
Additionally, the final figure presents in vitro slice physiology suggesting sex differences that are loosely connected to the behavioral findings (despite the authors in the abstract state: "..sex-specific differences in escape latency which aligned with our observation of higher basal spontaneous activity in male LHbCRF neurons"). However, without direct correlation or supporting analysis, this interpretation appears overstated and should be tempered.
Overall, I believe the manuscript requires major revision before being suitable for publication in Communications Biology. I hope the following detailed comments will help the authors improve their interesting study:

Major Points:

1. Figure 1:
A. It might strengthen the impact of Figure 1 to include representative images from additional brain regions such as the BNST, PVN,.....
B. In panel B, plotting individual mouse averages (e.g., as single dots) could provide a clearer view of variability across animals.
C. To further support the input analysis, the authors should consider including supplemental anterograde tracing using CRH-positive populations known to project to the LHb (e.g., CeA, BNST). This could help clarify input specificity and address challenges in identifying injection sites in the current images.

2. Figures 2–4:
A. Adding quantification throughout these figures—for instance, reporting the proportion of LHb neurons that are CRF-positive—could make the data more interpretable and impactful.
B. For the anterograde tracing, the use of a synaptophysin-tagged virus (e.g., AAV-DIO-Synaptophysin, as in Molas et al., 2023, eNeuro) might help distinguish axonal terminals from passing fibers and enhance anatomical specificity.
C. It may be informative to explore whether LHbCRF neurons send collaterals to multiple targets. This could be particularly helpful in interpreting the DREADD experiments and determining whether these cells operate uniformly or form output-

defined subpopulations.

3. DREADD experiments:
A. Clarifying the rationale for choosing excitatory rather than inhibitory DREADDs would help the reader. The authors might consider elaborating on this point earlier in the introduction, potentially building more directly on their previous findings and refining the focus to align closely with the study's main objectives.
B. It could be beneficial to provide more justification for the behavioral tests selected, perhaps linking them more explicitly to the study's central questions.
C. Some of the conclusions, such as the statement in the abstract ("LHbCRF neurons promote passive defensive responses..."), may benefit from more cautious phrasing. As there is no direct evidence of recruitment during behavior, framing these findings as suggestive rather than definitive may be more appropriate. Incorporating a loss-of-function experiments (inhibitory DREADD for instance) might help establish a stronger causal relationship.

---

4. Figure 7 (Looming Test):
A. Including speed analyses for both escape and action-locking behaviors could provide a richer understanding of the behavioral phenotype.
B. Evaluating whether the duration of action-locking is affected might offer additional insights into the nature of the observed responses.

---

5. Slice Physiology and Circuit Function:
The authors might consider testing intra-LHb connectivity, as this could reveal important aspects of local CRF system function and uncover potential recurrent circuitry within the LHb.

Reviewer #2

(Remarks to the Author)
This manuscript sought to anatomically map sources of CRF to the LHb and determine the effects of different LHb CRF sources in affective regulation, including anxiety-like behavior and responses to predator threat. Both the anatomical and behavioral findings are of interest to researchers studying the LHb and are a valuable resource. In particular, the discovery of a local population of CRF+ neurons in the LHb is highly novel and, in combination with the group's prior studies, highlights LHb modulation by both intra and extra-LHb CRF release. While these data are interesting and important for the field, there are numerous methodological issues with how these studies were performed that temper enthusiasm for the manuscript. These issues are related to proper control groups for DREADD studies, poor image quality, conclusions not supported by the data in its current form, and lack of evidence for anatomical specificity of LHb viral injections.

Issues:
- Why were two different DREADD constructs used for behavioral experiments? Why were two different mouse lines used for behavioral experiments? These should be standardized across experiments as much as possible as they confound comparisons between behavioral effects. The authors should give the rationale/reason for this discrepancy at very least.
- Related to the DREADD experiments, the authors included no mCherry control group, which is critical to evaluate any off-target effects of the JHU compound. This is especially important as JHU has been shown to modulate anxiety independently of DREADD expression in recent studies (see https://pmc.ncbi.nlm.nih.gov/articles/PMC10527408/)
- The viral-mediated tracing experiments seemed meant to detect sex differences in CRF inputs and outputs from the LHb. However, for the anterograde experiments the authors used AAV8-DIO-mcherry in males and AAV5-DIO-CHR2-YFP in females. The authors' claim that males and females appear to have similar strength of outputs from these neurons is confounded by this viral discrepancy, as is the fact that this claim is not supported by any quantification of axonal projections or attempts to normalize axonal projection density based on the number of neurons expressing virus in the LHb.
- The LHb in mice is an extremely small structure that can be difficult to accurately target with viral injections. Although the authors used a very small volume of virus, there is still a distinct possibility that viral expression spread beyond the LHb, namely to the PVT and the Hippocampus. The viral expression images that the authors show often do not even show the entire LHb, let along the PVT or the Hippocampus. Also, there are no images provided of bilateral LHb viral expression from the DREADD experiments; further, the paper provides coronal images of viral expression for all figures but changes to sagittal in the DREADD figure, why? It is much more difficult to ascertain viral spread using these types of slices, especially because there is no DAPI counterstain to see anatomical landmarks. Properly zoomed-out images that clearly show little to no viral expression outside of the LHb are critical for assessing the specificity of the paper's manipulations. Please add these for all viral experiments.
- The authors propose that the effects of Gq LHbCRF activation in males differ from females due to a higher baseline spontaneous activity of these neurons in males. If this is the case, might the LHbCRF neurons need to be driven more strongly by the DREADD actuator to get the behavioral effects seen in males with Looming Disc escape latency?
- The authors repeatedly claim that because LHbCRF neurons express vglut2 and not vGAT, they must be excitatory neurons. However, so far the most predominant population found to locally inhibit LHb glutamate neurons is the GAD2+/vGlut2+/vGAT- population. These cells are located in the same area as the LHbCRF neurons, but GAD was not stained for in Figure 2. Therefore, the authors should not and cannot conclude that their neurons are definitively excitatory. In order to address this possibility, the authors should perform ISH for CRF, GAD2, and vGlut2 in the LHB and actually provide adequate quantification of the overlap in both sexes.
- Why was retrograde tracing done with sexes collapsed whereas behavior and retrograde tracing were separately analyzed? This is a missed opportunity to compare inputs and outputs between sexes.

- Also related to figure 2: the images are extremely poor quality and very faint, with little ability to make out LHb structures or clearly note cell locations within the entire LHb. These images need to be brighter, more zoomed out, and shown in both sexes.
- What subregion of the LHb are CRF neurons in? From Figure 1 it appears they are medial, in the exact location where LHb GAD2 neurons generally are. But in Figure 3, they appear in the lateral region. Having more zoomed out images so that the entire LHb can be seen is important. It is also important to provide more quantification to characterize these cells in the LHb. What percentage of total LHb cells are they? There seem to be very few neurons expressing viral reporters in CRF cells in the tracing studies, but there are a large number of cells seen in the DREADD viral expression image in Fig 5. Please provide clarification on this.

Reviewer #3

(Remarks to the Author)
In this work of Flerlage and colleagues, from the Nugent lab, the authors identify a population of CRF-positive neurons in the lateral habenula. They determine that these cells are a subset of the major cell type in the LHb, namely glutamatergic cells. They also show that they project to various downstream targets, and possibly locally within the LHb itself. They chemogenetically activate these cells in the context of various anxiety/valence related behavioral assays. They show an absence of effects on various anxiety measures, but do show an increase in freezing behavior / action locking in a looming test. They also address potential sex differences in these LHbCRF neurons. For instance, describing differences in ex vivo firing patterns of the LHbCRF neurons between males and females. Overall the work is certainly of interest but there are a couple of points that would need to be addressed to bolster some of the conclusions.

Major:
1. Is there indeed an LHb population of cells that locally releases CRF?
The authors conclude that there is an LHbCRF population that projects locally within the LHb. While the authors indeed provide multiple lines of evidence for the existence of an LHbCRF population, it is not definitely demonstrated that these cells indeed project within the LHb. Current evidence for this is based on 2 experimental lines, but neither is conclusive.
i. The retrograde strategy of Figure 1. There the authors use a cre-dependent retrograde tracer that they inject into the LHb of CRF-Cre mice to directly map CRF+ inputs to the LHb. They find several brain regions with traced cells, including PAG, PVT and LHb itself. There are some issues however that prevent fully confident interpretation of this finding to support local projection. First, since there is no marker of the injection site, it’s hard to know whether indeed the LHb itself was exclusively hit across mice. So it is unclear where those fluorescently targeted LHb neurons project to (e.g. in LHb or in a perhaps surrounding structure also hit by the virus). Indeed as the authors also remark, it was surprising to find the PVT as an input. It may indeed be a real input, but it could also be cells picked up due to viral spread beyond LHb.

ii. The other line of evidence is anterograde (e.g. Figure 3 and 4). Here the LHb is targeted, in CRF-cre mice, with a cre-dependent anterograde virus. The authors observe fluorescence, including likely of neurites, in LHb. Though here it’s unknown if these contain synaptic endpoints so it is again unclear if they represent release sites. Also here PVT shows some fluorescence, but it is also unclear here whether this is an anterograde release site, or for instance cue to fluorescent cell bodies of PVTCRF neurons, targeted due to viral spread to such a nearby region.
To support the claim that LHbCRF neurons project within the LHb, additional evidence would be required. For instance, functional connectivity within the LHb, and/or forms of anatomical tracing that also show convergence of labeled neurites with markers for release sites.

2. Further characterization of location of LHbCRF neurons.
The existence of LHbCRF neurons is interesting, and the field would benefit from a better understanding of where these cells exactly are in LHb and how numerous they are. Regarding position: Some figures suggest a rather medial clustering within the LHb, though other figures do not necessarily seem to report the same. I was surprised to find little if any reflection on this potentially interesting point. The paper would benefit from a dedicated analysis of the position within the LHb of the LHbCRF cells.
Regarding number: It remains a bit unclear how big or small this population is. In some figures (e.g. Figure 2 and 3) the amount of LHbCRF cells seems to be very low. Though again, in other figures this looks a bit different. Figure 2 is also missing quantification in general. There are statements in the text about this Figure, such as that 100% of neurons are vglut2+ and that “only a small minority” has VGAT, but there is no information on the numbers behind these results as far as I can tell. What was the number of mice used here? The number of cells evaluated, with which number/percentage VGAT positive, etc?
These quantifications of position and number should ideally be done separately for the RNA scope data and for an experiment where those cells were instead virally captured, thereby also giving some insight in whether those two independent ways of showing the cells indeed converge on (within reason) a similar result and on the same population.

3. In general the microscopy images for histology can be improved in some regards.
Somewhat linked to point 2 above, in many figures the paper would benefit from seeing more of the LHb than currently the case. In Figure 1 we do not see the lateral extremities of the LHb, making it hard to judge if there are retrolabeled cells there or not. In figures 2 and 3 instead we see but a small fragment of the LHb, missing most of the ventral part. Figure 5 suddenly opts for sagittal representation of the LHb. Not inherently a problem, but of course making it difficult to see where along the medial-lateral axis we are, which may be, as described above, a very relevant factor. Figure 6 images could benefit from labelling of regions for easier understanding of positioning.

4. Validity of statistical analysis approach.
The authors use relatively low N experiments in many of the figures. For instance a lot of the tracing studies are done with n=3, and then it is also not entirely clear how the variability between those n=3 was. For instance in Figure 1, though it also applies to other figures, it's not clear if this distribution of found percentages was consistent across animals or not. This should be clarified.
Most importantly, for the main behavioral test where an effect is found (action locking / freezing) some questions arise about the statistical approach taken. It is not entirely clear to me whether here the N used represents trial number, or animal. It would seem to be trial number. In my view animal should be the independent unit on which the statistical analysis should be run for this experiment. Whether this is the case would need to be clarified, and if it is not done on animal then it would likely need to be reanalyzed in that way.

5. Female vs Male
I appreciate that the authors address potential differences between females and males in the behavioral tests. What is aside from that rather striking however is that Figure 3 (males) vs Figure 4 (females) seem to yield a very different situations for LHbCRF neurons, at least in the representative images shown. It is unclear whether (if this optical difference is indeed representative) this is due to differences in virus (AAV8-DIO-mCherry vs AAV5-EF1a-ChR2-EYFP) or in a difference between males and females. The authors need to clarify whether between males and females the LHbCRF populations are similar or different in their size/position. This to complement their functional analysis in Figure 8.

6. Are there reported effects of stimulation of LHbCRF neurons mediated by CRF?
Finally, it remains unclear whether any of the observed or inferred effects of LHbCRF neurons (e.g. local or distal connectivity, action locking) involve any CRF signaling. Since these are vglut2+ neurons that are also positive for CRF, a potentially dual mode of action is possible for these cells. There are currently no hints as to what the contribution of CRF is. An inclusion of an experiment addressing whether CRF is actually released and relevant for the role of these neurons, would be a strong added value to the work.

Minor point

1. Line 126 – 128: The sentence: "Notably, retrograde labeling was not detected in some other known CRF-expressing regions that project to the LHb, such as the VTA and CeA, supporting the rgAAV tracer's specificity" (similar argument in L267 and beyond) I find confusing.

In my view this should not be cited as evidence for specificity of the tracer. It's not an actual negative control, because we don't know if those CRF+ VTA and CeA cells indeed shouldn't project to LHb. In the meantime we know that retro AAV generally has considerable problems of tropism, labelling certain pathways much better than others. So it is very possible that these inputs do actually exist, but they are just not targetable via rg-AAV. That could also be viewed as a form of specificity, but probably not in the typical sense of the word, which is that the strategy would be specific if it doesn't have any off-target labelling (of non-CRF neurons). These sentences should therefore be amended. Moreover, the discussion should more clearly discuss the potential limitation of viral tropism giving a limited view on potential CRF inputs to LHb (in Fig 1) due to the sole use of retroAAV for retrograde mapping there.

Version 1:

Reviewer comments:

Reviewer #1

(Remarks to the Author)
In this revised version of the manuscript, the authors provide an outstanding amount of new data and analysis that significantly improves the quality of the work and strengthens its main conclusions. I sincerely appreciate the effort the authors have invested in addressing the revisions. They have fully and convincingly addressed my main concerns, and I therefore fully support the publication of this manuscript in its current form in Communications Biology.

Reviewer #3

(Remarks to the Author)
I appreciate the considerable efforts taken by the authors. In particular, the addition of functional patch-clamp data to demonstrate local connectivity made by the LHb-CRF neurons. Moreover, histological images and analyses have improved a lot, making the study much more informative. Similarly, statistical analyses and data representations have improved considerably. I agree with the authors that the topic of co-release of CRF can be an interesting follow-up topic for study. Overall, my main points have been dealt with well and I have no further concerns. - Frank Meye

Open Access This Peer Review File is licensed under a Creative Commons Attribution 4.0 International License, which permits use, sharing, adaptation, distribution and reproduction in any medium or format, as long as you give appropriate credit to the original author(s) and the source, provide a link to the Creative Commons license, and indicate if changes were made.
In cases where reviewers are anonymous, credit should be given to 'Anonymous Referee' and the source.
The images or other third party material in this Peer Review File are included in the article's Creative Commons license, unless indicated otherwise in a credit line to the material. If material is not included in the article's Creative Commons license and your intended use is not permitted by statutory regulation or exceeds the permitted use, you will need to obtain permission directly from the copyright holder.
To view a copy of this license, visit https://creativecommons.org/licenses/by/4.0/

Response to Reviewers

We would like to thank all three reviewers for their positive feedback and enthusiasm for this work. We very much appreciate the constructive and valuable comments and suggestions. We have revised the manuscript accordingly and provided files with track changes as well as clean copies of the main manuscript.

For the convenience of reviewers, we outline the major changes made in the revision that address the concerns of our reviewers. Based on excellent suggestions from our reviewers, we performed new experiments for RNAscope, anterograde strategy, and optogenetic experiments. The results are now incorporated in the revised manuscript, including a new anterograde tracing study using ChrimsonR in both male and female CRH-Cre::Ai6 mice and new microscopy using a Zeiss AxioScan. This anterograde strategy addressed the issue of our initial two strategies (mCherry and ChR2) used in CRH-Cre male and female mice.

Of note, we utilized this new transgenic mouse line (CRH-Cre::Ai6) not only to verify our targeting of the LHb and confirmation of ChrimsonR expression, but also to take advantage of the red-shifted opsin's superiority in penetrating deeper tissues for circuit mapping (Lin et al., 2013; Goyer & Roberts, 2020). This strategy was similarly used to perform an optogenetic study for the dissection of local circuits within the LHb arising from Chrimson-expressing LHb^{CRF} neurons that innervated non-CRF LHb neurons; we utilized the GFP labeling of CRF neurons to distinguish between CRF+ and CRF- populations.

We have also performed new RNAscope using vGLUT2, vGAT, and GAD2 (the latter added in response to a reviewer suggestion) probes in CRH-Cre::Ai14 mice for molecular characterization. Initially, we attempted to include a *Crh* probe; however, after extensive troubleshooting and communication with ACD, we encountered significant non-specific binding where the *Crh* probe labeled almost the entire LHb and negative control regions like the mHb. Consequently, we utilized the td-Tomato reporter in the Ai14 line to reliably identify CRF+ neurons.

We would like to address our delay in providing this revision. As employees of a federal university, we were impacted by two government shutdowns, which negatively affected animal breeding, supply ordering, and operations. We hope that with the inclusion of this extensive new work and statistical analysis, the reviewers will be satisfied with the revision. Below, we now address all the concerns point-by-point for each reviewer.

Reviewers' comments:

Reviewer #1 (Remarks to the Author):

In this ms, Flerlage, Gouty, Thomas and colleagues identify for the first time a population of CRF-expressing neurons in the lateral habenula (LHb). Using anatomical tracing, viral approaches, and CRH-Cre mouse lines (CRH-Cre and

CRH-Cre:Ai14), the authors describe the input–output architecture of this CRF system in the LHb. They further employ a “gain-of-function” strategy, expressing excitatory DREADDs in LHbCRF neurons to investigate their potential roles in various behaviors, including locomotion, anxiety, reward processing, and innate fear. Notably, they observe altered behavioral strategies in the looming test (increased action-locking) and a sex-dependent difference in escape latency following agonist injection.
This work has the merit of highlighting a previously overlooked LHb neuronal population, and the inclusion of both sexes is a valuable strength. However, the study feels preliminary in several key aspects, and some conclusions are not sufficiently supported by the data. For instance, the first four figures lack quantification and rely only on representative images— a limitation when characterizing a novel neuronal population. Furthermore, the rationale for using a gain-of-function approach over a loss-of-function is not clearly explained, particularly given the causal interpretations drawn from the DREADD experiments. A functional description of these neurons under physiological conditions is also missing.
Additionally, the final figure presents in vitro slice physiology suggesting sex differences that are loosely connected to the behavioral findings (despite the authors in the abstract state: "..sex-specific differences in escape latency which aligned with our observation of higher basal spontaneous activity in male LHbCRF neurons"). However, without direct correlation or supporting analysis, this interpretation appears overstated and should be tempered.
Overall, I believe the manuscript requires major revision before being suitable for publication in Communications Biology. I hope the following detailed comments will help the authors improve their interesting study:

Response: We would like to sincerely thank R1 for their rigorous analysis of our work and their positive feedback on the value of this work. We apologize for failing in the initial submission to provide more comprehensive anatomical tracing studies. We used a combination of Allen brain atlas as well as Paxinos to quantitatively reanalyzed our retrograde tracing studies as well as performed new RNAscope studies with similar strategy in male and female mice. However, given our limited number of transgenic mice due to breeding issues with government shutdown and animal ordering from JAX, in new anterograde studies with ChrimsonR-tdTomato (n=4, 2 males and 2 females), we only provide the representative images with higher quality showing the projection areas for LHb^{CRF} neurons. We utilized ChrimsonR because its red-shifted excitation spectrum offers superior tissue penetration and reduced light scattering compared to blue-light-activated ChR2, making it significantly more robust for mapping distal terminals [1,2]. We then moved the initial anterograde imaging by mCherry and ChR2 with enhancement of fluorescent signals to supplemental file. Of note, we were unable to provide larger scale images for the previous anterograde tracing studies with these two strategies given that most of the signal was faded after two years in these sections. But we hope you will be

satisfied with our new anterograde Chrimson tracing study, re-quantification of retrograde tracing data as well as our new RNAscope with quantification as well as new optogenetic data based on your excellent suggestion that also addressed some of the physiological differences in LHb^{CRF} neurons in male and female mice.

Major Points:

1. Figure 1:
A. It might strengthen the impact of Figure 1 to include representative images from additional brain regions such as the BNST, PVN,.....

Response: We thank you again for this excellent suggestion. As mentioned above we re-analyzed and quantified this data using a combination of both Allen and Paxinos atlases and larger image sections and found interesting new regions that also provide CRF inputs to the LHb. We included the major LHb-projecting CRF inputs as in Figure 1 with both larger scale images with overlayed atlas and provided higher magnification images, adding their corresponding Bregma/ AP levels and provided images for other CRF inputs that were less prevalent but still existed in Supplementary Figure 1.

B. In panel B, plotting individual mouse averages (e.g., as single dots) could provide a clearer view of variability across animals.

Response: We have now designated individual symbols to illustrate this variability.

C. To further support the input analysis, the authors should consider including supplemental anterograde tracing using CRH-positive populations known to project to the LHb (e.g., CeA, BNST). This could help clarify input specificity and address challenges in identifying injection sites in the current images.

Response: To address this issue, we have performed new anterograde tracing using Cre-dependent expression of ChrimsonR-tdTomato in CRH-Cre::Ai6 mice which enabled us to show the exclusive expression of ChrimsonR-tdTomato only in LHb^{CRF} neurons that are labeled by GFP (Figures 5-6 and supplementary figures 8 and 9). In all of our injections, we always verify targeting which showed no off-target expression of ChrimsonR in any CRF-expressing region including CeA, BNST and PVN. Further we completely show the high and low magnification images of the LHb injection sites which verify our success in targeting exclusively and selectively the LHb. This new experiment has been more robust in identifying brain regions that LHb^{CRF} neurons project to but also were consistent with our two other strategies in finding LHb itself, RMTg, SNc and PAG as some of the projection targets of these neurons.

2. Figures 2–4:

A. Adding quantification throughout these figures—for instance, reporting the proportion of LHb neurons that are CRF-positive—could make the data more interpretable and impactful.

Response: We have now provided this quantification in Figure 4. We used an unbiased strategy of counting all CRF+ neurons and transcripts in RNAscope study within the LHb perimeter by tracing the borders of LHb using the atlas as shown in Supplementary Figure 3 where we statistically analyzed whether the size of LHb show sex differences in 5 AP levels which was not the case. Therefore, we included all the CRF neurons counted within LHb borders and quantified the number of these neurons and the expression of VGLUT2, vGAT and GAD2 in CRF+ neurons identified by td-Tomato expression. Interestingly male mice showed higher number of CRF+ neurons that are all also VGLUT2 positive, but we also found that a minor subpopulation of CRF neurons co-express vGAT and VGLUT2 that are negative for GAD2, which was also higher in males than females. These data are now documented in multiple figures for RNAscope (Figures 2–3 and Supplementary Figures 3–7).

B. For the anterograde tracing, the use of a synaptophysin-tagged virus (e.g., AAV-DIO-Synaptophysin, as in Molas et al., 2023, eNeuro) might help distinguish axonal terminals from passing fibers and enhance anatomical specificity.

Response: We appreciate the R1's suggestion but our strategy using ChrimsonR-tdTomato in CRH-Cre::Ai6 mice has been also successful in tracing of these terminals in multiple structures where our initial strategies failed to reveal. Additionally, our optogenetic strategy also verified the local connection within the LHb itself with non-CRF neurons. Overall, ChrimsonR is generally considered superior to ChR2 for deep-tissue terminal labeling and circuit mapping given its red-shifted spectrum [1,2].

C. It may be informative to explore whether LHbCRF neurons send collaterals to multiple targets. This could be particularly helpful in interpreting the DREADD experiments and determining whether these cells operate uniformly or form output-defined subpopulations.

Response: These are very excellent ideas, and we also consider exploring these using projection-specific mappings and the use of intersectional DREADD strategies[3] to dissect distinct LHb^{CRF} circuits, but we hope that our R1 appreciates that this is a huge undertaking beyond the scope of this work which focuses on the characterization of these novel subpopulation.

3. DREADD experiments:

A. Clarifying the rationale for choosing excitatory rather than inhibitory DREADDs would help the reader. The authors might consider elaborating on this point

earlier in the introduction, potentially building more directly on their previous findings and refining the focus to align closely with the study's main objectives.

Response: We completely agree with the reviewer's assessment. Our previous work in both mice and rats [4-6] established the LHb as a highly CRF-responsive region. In the current study, we identify a novel subpopulation of LHb neurons that express CRF, and our optogenetic and tracing data confirms they form both local intra-LHb circuits and widespread long-range projections. The decision to utilize a gain-of-function (Gq-DREADD) strategy was driven by the structural and functional diversity of these projections. Our anterograde mapping revealed LHb^{CRF} targets across multiple neuroanatomical axes, including: Hypothalamic/Diencephalic targets: Lateral hypothalamus (LH) and zona incerta (ZI) at Bregma as shown in both sexes at -1.82mm, Midbrain defensive centers: Superior colliculus (SC) and periaqueductal gray (PAG) at Bregma -3.52mm, Monoaminergic nuclei: Ventral tegmental area (VTA) and substantia nigra pars compacta (SNc) at Bregma -3.52mm, Hindbrain/Raphe nuclei: Dorsal raphe (DR), rostromedial tegmental nucleus (RMTg), and median raphe (MnR) at Bregma -4.36mm (Figures 5-6).Given the established role of the LHb in stress-related avoidance and innate fear, activating this widespread LHb^{CRF} circuits allowed us to determine whether these specific neurons play a role in mediating behavioral responses to threat. Furthermore, previous research indicates that chemogenetic inhibition (Gi-DREADD) of major LHb projection targets—such as the VTA, DRN, or RMTg—often fails to alter baseline behaviors (locomotion, anxiety in OFT/LDT) unless the animal is specifically challenged by a stressor[3]. Thus, a gain-of-function approach was more appropriate for initial characterization. Interestingly, while the LHb is generally associated with aversion, activation of distinct subpopulations, such as PACAP-expressing neurons, has been shown to produce rewarding effects (CPP) and decrease fear[7]. Specifically, the VLST was chosen for the assessment of innate defensive behaviors, as hypothalamic CRF neurons and LHb neurons have been shown to play a significant role in the modulation of this behavior [8-11]. This further motivated us to test LHb^{CRF} activation in CPP as well as EZM, and the VLST. Our results demonstrate that LHb^{CRF} neurons selectively modulate innate defensive strategies—specifically enhancing "action-locking" behaviors—with notable sex differences in escape latencies, and shelter stay. We have now incorporated this detailed rationale into our Introduction and Discussion sections to better clarify our focus.

B. It could be beneficial to provide more justification for the behavioral tests selected, perhaps linking them more explicitly to the study's central questions.

Response: That's an excellent suggestion. Given the role of LHb in motivated behaviors, we included OFT, EZM, and LDT as controls to ensure activation did not simply increase general anxiety or affect locomotor activity. We tested whether activation was rewarding or aversive in CPP, as seen in other stress-neuropeptide

populations like LHb-PACAP[7]. Furthermore, LHb has been shown to mediate defensive behavior specifically in the loom-shadow test [10], prompting us to test this specific behavior. We have now incorporated this detailed rationale into both the Introduction and Discussion to more clearly justify our selection of these specific behavioral assays

C. Some of the conclusions, such as the statement in the abstract ("LHbCRF neurons promote passive defensive responses..."), may benefit from more cautious phrasing. As there is no direct evidence of recruitment during behavior, framing these findings as suggestive rather than definitive may be more appropriate. Incorporating a loss-of-function experiments (inhibitory DREADD for instance) might help establish a stronger causal relationship.

Response: We completely agree and rephrased this in the abstract. While we appreciate the excellent suggestion of inhibitory DREADDs, we think it will be important to include complementary silencing of LHb^{CRF} neurons in our future studies focusing on this subpopulation of LHb neurons in the context of stress and trauma paradigms.

4. Figure 7 (Looming Test):
A. Including speed analyses for both escape and action-locking behaviors could provide a richer understanding of the behavioral phenotype.

Response: We appreciate this insightful suggestion! While our current single-camera lateral orientation precluded accurate center-of-mass tracking for reliable speed quantification, we have significantly expanded our behavioral analysis to provide a more granular characterization of the defensive phenotypes. Based on your recommendation, we re-evaluated our Visual Looming Shadow Test (VLST) videos and identified three distinct behavioral outcomes: (1) escape, (2) action-locking, and (3) action-locking that transitions to escape in which mice exhibit an immediate freezing behavior followed by a delayed escape to the shelter. Our previous analysis had grouped the third category within general "escapes." By separating these, we confirmed our intimal observation that LHb^{CRF} activation significantly shifts the defensive profile toward action-locking in both sexes. Specifically, our new analysis (now presented in Figure 8) demonstrates: Chemogenetic activation significantly increased the frequency of trials resulting in action-locking across both males and females. LHb^{CRF} activation prolonged the duration of action-locking behaviors in both sexes, regardless of whether they eventually transitioned to an escape. We added an analysis of shelter stay duration as a measure of post-escape safety-seeking. We found that activation in females specifically resulted in a protracted shelter stay, suggesting heightened threat perception or delayed recovery compared to males[9]. Regarding the reviewer's point on speed, we are currently optimizing a multi-camera automated tracking system for future studies to capture this parameter. We believe these new, detailed behavioral classifications provide the "richer understanding" requested by the reviewer. All updated data and the revised figure are now included in the manuscript.

B. Evaluating whether the duration of action-locking is affected might offer additional insights into the nature of the observed responses.

Response: We thank the reviewer for this excellent suggestion! In response, we expanded our analysis of the VLST to include both the duration of the action-locking bouts and post-escape shelter behavior. Chemogenetic activation of LHb^{CRF} neurons significantly increased the overall duration of action-locking in both sexes. Furthermore, our analysis of "shelter stay" as a measure of post-escape safety-seeking revealed a notable sex difference. Gq-activation in females resulted in a significantly prolonged stay within the shelter compared to males, who returned to exploring the arena more rapidly. This increased occupancy in the shelter following the looming threat in females may indicate heightened fear levels, a persistent perception of a high-intensity threat, or a reduced sense of safety, all of which contribute to a longer latency to re-emerge into the arena. These new datasets and their implications for sex-specific defensive strategies have been incorporated into the Results and Discussion sections of the revised manuscript.

5. Slice Physiology and Circuit Function:
The authors might consider testing intra-LHb connectivity, as this could reveal important aspects of local CRF system function and uncover potential recurrent circuitry within the LHb.

Response: Another outstanding suggestion! We performed new optogenetic experiments showing local excitatory connections between LHb^{CRF} neurons and non-CRF LHb neurons (Figure 10). We detected sex differences where females showed stronger excitatory synaptic connections due to a higher probability of presynaptic glutamate release (lower PPR). We are hoping to use advanced strategies such as CRF GRAB sensors and intersectional DREADD strategies in future studies to demonstrate CRF-glutamate co-release.

Reviewer #2 (Remarks to the Author):

This manuscript sought to anatomically map sources of CRF to the LHb and determine the effects of different LHb CRF sources in affective regulation, including anxiety-like behavior and responses to predator threat. Both the anatomical and behavioral findings are of interest to researchers studying the LHb and are a valuable resource. In particular, the discovery of a local population of CRF+ neurons in the LHb is highly novel and, in combination with the group's prior studies, highlights LHb modulation by both intra and extra-LHb CRF release. While these data are interesting and important for the field, there are numerous methodological issues with how these studies were performed that temper

enthusiasm for the manuscript. These issues are related to proper control groups for DREADD studies, poor image quality, conclusions not supported by the data in its current form, and lack of evidence for anatomical specificity of LHb viral injections.

We sincerely thank you for your positive feedback on the importance of this study as well as your valid criticism and excellent suggestions that helped us to improve this work.

Issues:
- Why were two different DREADD constructs used for behavioral experiments? Why were two different mouse lines used for behavioral experiments? These should be standardized across experiments as much as possible as they confound comparisons between behavioral effects. The authors should give the rationale/reason for this discrepancy at very least.

Response: We completely agree with the reviewer regarding the importance of standardization. To address this, we have moved the behavioral data obtained using the Gq-DREADD-mCherry strategy in CRH-Cre mice to Supplementary Figure 10. We have centralized the primary results in Figures 7 and 8 using a standardized approach in both sexes: CRH-Cre::Ai14 mice injected with HA-Gq-DREADD-mCitrine. The rationale for utilizing CRH-Cre::Ai14 or CRH-Cre::Ai6 reporter lines in conjunction with the standard CRH-Cre line was to facilitate the specific identification and visualization of CRF+ neurons for our RNAscope, electrophysiology, and local circuit mapping experiments. It is important to note that all three lines utilize the same Cre-driver to target the CRF population; the reporter lines (Ai14/Ai6) simply offer the technical advantage of endogenous fluorophore expression (tdTomato or ZsGreen) upon Cre-mediated recombination. Extensive literature has validated that these Cre-dependent reporter strains reliably recapitulate the expression patterns of the underlying Cre-driver line without altering the physiological or behavioral characteristics of the target neuronal population [12,13]. Specifically, the CRH-Cre line has been widely demonstrated to target CRF neurons with high specificity and efficiency across various brain regions, and the addition of the Ai reporter alleles does not introduce confounding variables in behavioral responses[14-18]. By transitioning our main behavioral figures to the CRH-Cre::Ai14 cohort, we have ensured a more uniform comparison across sexes and experiments while maintaining the necessary labeling for our physiological and tracing studies. We have now clarified this rationale and the consistency of the Cre-mediated targeting in the revised Materials and Methods and Results sections.

- Related to the DREADD experiments, the authors included no mCherry control group, which is critical to evaluate any off-target effects of the JHU compound.

This is especially important as JHU has been shown to modulate anxiety independently of DREADD expression in recent studies (see https://pmc.ncbi.nlm.nih.gov/articles/PMC10527408/)

Response: We appreciate the reviewer's concern and are indeed aware of the recent findings regarding potential off-target effects of DREADD agonists at high doses (specifically JHU37160 at 1mg/kg used in male rats in this study)[19]. However, we have several lines of evidence supporting the specificity of our findings in mice. In our previous work [20], we utilized a control virus (mCherry) group with both saline and JHU37160 injections and found no agonist-induced behavioral alterations. For the current study, we performed pilot control experiments using mCherry-only injections in the LDT, which confirmed that JHU37160 did not alter locomotor activity or baseline anxiety-like behaviors in male or female rats. Crucially, throughout the current study, Gq-DREADD-expressing mice administered JHU37160 showed no significant differences in general motor activity or anxiety-like metrics in the OFT, LDT, or EZM compared to saline controls. These baseline assays serve as rigorous internal controls for our VLST results, demonstrating that the observed shifts in defensive strategies are not a byproduct of induced motor deficits or generalized anxiety. Furthermore, we utilized JHU37160 at a low dose (0.3 mg/kg), a concentration shown to possess high potency and selectivity for DREADDs with minimal off-target effects on reward-seeking or locomotion in mice[21]. By employing this specific dosage, we have effectively mitigated the concerns associated with non-specific behavioral modulation reported at higher concentrations.

- The viral-mediated tracing experiments seemed meant to detect sex differences in CRF inputs and outputs from the LHb. However, for the anterograde experiments the authors used AAV8-DIO-mcherry in males and AAV5-DIO-CHR2-YFP in females. The authors' claim that males and females appear to have similar strength of outputs from these neurons is confounded by this viral discrepancy, as is the fact that this claim is not supported by any quantification of axonal projections or attempts to normalize axonal projection density based on the number of neurons expressing virus in the LHb.

Response: We acknowledge that our initial strategies using different viral constructs (mCherry vs. ChR2-YFP) were not ideal for a direct comparison of anterograde LHb^{CRF} projections. To address this, we transitioned to a standardized strategy using AAV5-Syn-FLEX-ChrimsonR-tdTomato in CRH-Cre::Ai6 mice of both sexes. This approach utilized superior tissue penetration and reduced scattering properties of red-shifted opsins [1,2], providing a much more robust platform for terminal mapping than our previous methods. The use of the Ai6 reporter line allowed us to validate our targeting specificity by confirming that ChrimsonR expression was restricted to GFP-labeled CRF+ neurons within the LHb. Utilizing high-resolution Zeiss AxioScan microscopy, this standardized approach allowed us to identify several key projection targets, including

the RMTg, SNc, and PAG, which were consistently observed across both sexes and further validated the findings of our initial viral strategies. Unfortunately, as mentioned previously, significant breeding and supply disruptions caused by government shutdowns limited our available cohort for this specific mapping study to n=4 (2 males, 2 females), which precluded a meaningful statistical quantification of sex differences in projection density. Consequently, we have tempered our conclusions; we no longer claim a lack of sex differences in output strength but rather characterize the fundamental neuroanatomical targets of this novel population. We hope to employ intersectional strategies (e.g., Flp-Cre) in future studies to provide a fully quantified, normalized map of these distinct LHb^{CRF} circuits.

- The LHb in mice is an extremely small structure that can be difficult to accurately target with viral injections. Although the authors used a very small volume of virus, there is still a distinct possibility that viral expression spread beyond the LHb, namely to the PVT and the Hippocampus. The viral expression images that the authors show often do not even show the entire LHb, let along the PVT or the Hippocampus. Also, there are no images provided of bilateral LHb viral expression from the DREADD experiments; further, the paper provides coronal images of viral expression for all figures but changes to sagittal in the DREADD figure, why? It is much more difficult to ascertain viral spread using these types of slices, especially because there is no DAPI counterstain to see anatomical landmarks. Properly zoomed-out images that clearly show little to no viral expression outside of the LHb are critical for assessing the specificity of the paper's manipulations. Please add these for all viral experiments.

Response: We apologize for not providing such images initially. We carefully control for targeting and have now included low and high magnification AxioScan images showing selective targeting of the LHb and the absence of spread into the PVT or Hippocampus.

- The authors propose that the effects of Gq LHbCRF activation in males differ from females due to a higher baseline spontaneous activity of these neurons in males. If this is the case, might the LHbCRF neurons need to be driven more strongly by the DREADD actuator to get the behavioral effects seen in males with Looming Disc escape latency?

Response: This is a profound observation, and we have now incorporated the possibility of a differential recruitment threshold and homeostatic regulation into our revised Discussion. Our data suggests that males and females may employ divergent cellular and synaptic strategies to modulate defensive behaviors. If female LHb^{CRF} neurons possess a lower baseline firing rate, as our electrophysiological data suggests, they may indeed require a more robust "fold-change" in activity to reach the threshold necessary to alter rapid, time-sensitive behaviors like escape latency. Interestingly, this

lower intrinsic excitability in females may be a homeostatic compensatory response to their significantly stronger local synaptic drive. We found that LHb^{CRF} neurons in females form more potent local excitatory connections onto non-CRF LHb neurons, characterized by a higher probability of presynaptic glutamate release (lower PPR) compared to males. This enhanced local synaptic gain in females likely provides the physiological basis for their prolonged action-locking duration as well as longer duration of shelter stay—a post-escape behavior indicative of heightened fear and persistent threat perception[9]. Conversely, while individual neurons in males are more intrinsically excitable (Figure 9) and exist in higher absolute numbers (Figure 4), their local synaptic connections appear less efficient than those in females (Figure 10). This suggests that the behavioral effects in males may rely on a higher population-level output and higher baseline firing, whereas female responses are driven by high-gain synaptic transmission that may trigger intrinsic "dampening" to maintain circuit stability.

As we have not yet dissected the specific synaptic weights of LHbCRF projections to distal targets (e.g., PAG or RMTg), it is highly likely that these sex-dependent differences in baseline excitability, population size, and local synaptic gain dictate the distinct behavioral outcomes of chemogenetic manipulation. We have updated our Discussion to explore these complex homeostatic and circuit-specific mechanisms.

- The authors repeatedly claim that because LHbCRF neurons express vglut2 and not vGAT, they must be excitatory neurons. However, so far the most predominant population found to locally inhibit LHb glutamate neurons is the GAD2+/vGlut2+/vGAT- population. These cells are located in the same area as the LHbCRF neurons, but GAD was not stained for in Figure 2. Therefore, the authors should not and cannot conclude that their neurons are definitively excitatory. In order to address this possibility, the authors should perform ISH for CRF, GAD2, and vGlut2 in the LHB and actually provide adequate quantification of the overlap in both sexes.

Response: We thank the reviewer for this excellent and insightful suggestion. To address this possibility, we performed new multiplexed RNAscope in CRH-Cre::Ai14 mice using probes for vGAT, vGLUT2, and GAD2. Utilizing Zeiss AxioScan imaging and QuPath automated analysis, we have now provided a comprehensive quantification of the neurochemical profile and topographical distribution of LHb^{CRF} neurons across the rostrocaudal axis.

Neurochemical Profile and Functional Identity: Our analysis revealed that 100% of identified CRF+ neurons co-express *vGLUT2*. Furthermore, we found that approximately 17–18% of these neurons also co-express Gad2, while a negligible minority (2–5%, primarily in males) co-express vGAT and vGLUT2.

Crucially, while a GAD2+/vGlut2+/vGAT- population has been reported to provide local inhibition within the LHb as you correctly suggested [22], our functional optogenetic data (Figure 10) demonstrated that stimulation of LHbCRF neurons evoked purely glutamatergic oEPSCs in local non-CRF targets, with no detectable inhibitory component. This suggests that the subset of CRF neurons co-expressing GAD2 likely corresponds to a distinct excitatory class recently defined in the literature. Specifically, Quina et al. (2020) demonstrated that GAD2 expression in the LHb defines a specific class of excitatory neurons that serve as a glutamatergic output pathway to the mesopontine tegmentum[23]. Therefore, we conclude that the LHb^{CRF} population is functionally excitatory, and the GAD2-positive fraction likely contributes to long-range excitatory projections rather than local inhibition.

Topographical and Rostrocaudal Distribution: Our data shows that the highest density of LHb^{CRF} neurons occurs at Bregma -1.06 mm and -1.34 mm. The population remains present but begins to decrease at -1.58 mm and is significantly less prevalent at the most caudal levels analyzed (-1.94 and -2.18 mm). This rostral-heavy distribution is a hallmark of several specialized LHb subpopulations, including LHb GAD2+ neurons.

In our rostral sections (-1.06 to -1.34 mm), a significant cluster of LHb^{CRF} neurons is located in the medial part of the LHb (LHbM), bordering the medial habenula (mHb). This aligns with the classic "hotspot" for GAD2-expressing neurons in the LHb, which are known to concentrate medially. However, unlike some GAD2 populations that are strictly medial, our RNAscope (Figures 2–4 and Supplementary Figures 3–7) shows CRF neurons also extending into the lateral part of the LHb (LHbL). The GAD2+/vGlut2+/vGAT- excitatory population identified here (~17–18% of our CRF neurons) tends to sit in the oval and medial subnuclei, consistent with the known topography of excitatory GAD2 neurons [23].

We have now updated our Results and Discussion to include this comprehensive neurochemical quantification and have integrated these citations to provide a more detailed interpretation of the LHb^{CRF} neurotransmitter phenotype. Given that the majority of LHb^{CRF} neurons were localized to the rostral LHb (-1.06 to -1.34 mm), we have included one AP level (-1.06) in our main figures and provided the -1.34 and -1.58 mm levels in the Supplementary Information).

- Why was retrograde tracing done with sexes collapsed whereas behavior and retrograde tracing were separately analyzed? This is a missed opportunity to compare inputs and outputs between sexes.

Response: We completely agree with the reviewer that comparing inputs and outputs between sexes is a highly valuable pursuit. Unfortunately, due to the significant breeding and supply disruptions caused by the government shutdowns mentioned previously, we were limited by the number of available CRH-Cre mice for this specific retrograde tracing study (n=3; 2 males, 1 female). This sample size precluded the

performance of meaningful sex-specific statistical analyses for this particular dataset. To provide as much transparency as possible, we have updated Figure 1 and Supplementary Figure 1 to include individual data points for each mouse (designated by unique symbols), allowing for a clear view of the variability across the animals. While the retrograde tracing was collapsed due to these constraints, we prioritized sex-specific analysis in all other aspects of the revision where we possessed sufficient power. This includes our comprehensive RNAscope quantification (7 males, 6 females; Figure 4 and Supplementary Fig 3), our Visual Looming Shadow Test (VLST) behavioral assays (Figure 8), and our electrophysiology and optogenetic experiments (Figures 9-10). Together, these datasets identified significant and novel sex-dependent differences in LHb^{CRF} population size, synaptic strength, and defensive strategy selection. In our future studies, we hope to perform a more comprehensive, high-powered analysis of both CRF inputs and outputs using intersectional viral strategies to fully map potential sex-specific architectures in these circuits. We believe these current high-powered datasets address the reviewer's interest in sex-specific circuit function and provide a robust foundation for the study's conclusions.

- Also related to figure 2: the images are extremely poor quality and very faint, with little ability to make out LHb structures or clearly note cell locations within the entire LHb. These images need to be brighter, more zoomed out, and shown in both sexes.

Response: We apologize for the insufficient quality of the initial images. We have thoroughly revisited these experiments and utilized Zeiss AxioScan microscopy to provide high-resolution, large-scale mosaic images for both sexes. These new images feature enhanced fluorescence signals and are presented with overlaid brain atlas borders and DAPI counterstaining to clearly delineate the LHb substructures. This improved visualization allows for an unambiguous identification of cell locations across the entire extent of the LHb in both male and female mice.

- What subregion of the LHb are CRF neurons in? From Figure 1 it appears they are medial, in the exact location where LHb GAD2 neurons generally are. But in Figure 3, they appear in the lateral region. Having more zoomed out images so that the entire LHb can be seen is important. It is also important to provide more quantification to characterize these cells in the LHb. What percentage of total LHb cells are they? There seem to be very few neurons expressing viral reporters in CRF cells in the tracing studies, but there are a large number of cells seen in the DREADD viral expression image in Fig 5. Please provide clarification on this.

Response: We appreciate the opportunity to clarify the topography of this population. The perceived discrepancy in previous figures was due to a lack of representative images across the full rostrocaudal axis. Our new, comprehensive RNAscope analysis across 5 AP levels (Figures 2–4 and supplemental Figures 3-7) reveals that the majority

of LHb^{CRF} neurons are localized to the rostral LHb (Bregma -1.06 to -1.34 mm). In these rostral sections, a significant cluster of CRF+ neurons is located in the medial part of the LHb (LHbM), bordering the medial habenula (mHb). This area indeed overlaps with the classic "hotspot" for GAD2-expressing neurons. However, our new high-resolution images show that the CRF+ population is not strictly medial; it extends into the lateral part of the LHb (LHbL) as well. This distribution pattern, predominantly rostral and medial but extending laterally, is consistent across our different AP levels. To ensure our quantification was robust and unbiased, we performed a detailed volumetric analysis of the LHb surface area across all 5 AP levels (Supplementary Figure 3). We found no significant differences in LHb surface area between male and female mice at any rostrocaudal level. This confirm that the higher absolute number of CRF+ neurons observed in males (Figure 4) reflects a true difference in neuronal density rather than a difference in the overall size of the structure. Overall, we have provided an unbiased quantification of the total number and percentage of these neurons. LHb^{CRF} neurons represent a discrete but significant subpopulation, with males possessing a higher absolute number of CRF+ neurons compared to females.

Reviewer #3 (Remarks to the Author):

In this work of Flerlage and colleagues, from the Nugent lab, the authors identify a population of CRF-positive neurons in the lateral habenula. They determine that these cells are a subset of the major cell type in the LHb, namely glutamatergic cells. They also show that they project to various downstream targets, and possibly locally within the LHb itself. They chemogenetically activate these cells in the context of various anxiety/valence related behavioral assays. They show an absence of effects on various anxiety measures, but do show an increase in freezing behavior / action locking in a looming test. They also address potential sex differences in these LHbCRF neurons. For instance, describing differences in ex vivo firing patterns of the LHbCRF neurons between males and females. Overall the work is certainly of interest but there are a couple of points that would need to be addressed to bolster some of the conclusions.

We thank R3 for their positive feedback and truly appreciate their valuable comments that helped us improve this manuscript.

Major:
1. Is there indeed an LHb population of cells that locally releases CRF?
The authors conclude that there is an LHbCRF population that projects locally within the LHb. While the authors indeed provide multiple lines of evidence for the existence of an LHbCRF population, it is not definitely demonstrated that these cells indeed project within the LHb. Current evidence for this is based on 2 experimental lines, but neither is conclusive.

Response: This is an excellent point, and we have taken significant steps to provide functional evidence for this local circuit. We have now demonstrated via optogenetics that LHb^{CRF} neurons indeed project within the LHb and form functional excitatory synaptic connections with non-CRF LHb neurons (Figure 10). By recording from GFP-negative neurons while selectively stimulating ChrimsonR-expressing CRF+ neurons, we observed robust oEPSCs, confirming a direct intra-habenular glutamatergic microcircuit.

Regarding the specificity of our targeting, we have now provided high-resolution, zoomed-out AxioScan images that clearly delineate the selective targeting of the LHb. We utilize small injection volumes to ensure viral spread is strictly restricted to the LHb perimeter, a precision that has been verified across all experimental cohorts. Furthermore, the reviewer raises an outstanding question regarding whether these neurons locally release the CRF peptide itself. Due to several obstacles and limitations including the restricted number of available mice following the government shutdowns, we have been unable to perform the complex experiments required to definitively prove peptide release, such as high-frequency optogenetic stimulation combined with CRF GRAB sensors or the use of floxed-CRH mice. While we find this question of utmost importance and intend to pursue it in future studies, we believe the current functional data demonstrating glutamate release significantly advances our understanding of this novel population. We hope the reviewer appreciates that the depth of new physiological and anatomical data provided in this revision significantly strengthens the impact and validity of our claims.

i. The retrograde strategy of Figure 1. There the authors use a cre-dependent retrograde tracer that they inject into the LHb of CRF-Cre mice to directly map CRF+ inputs to the LHb. They find several brain regions with traced cells, including PAG, PVT and LHb itself. There are some issues however that prevent fully confident interpretation of this finding to support local projection. First, since there is no marker of the injection site, it's hard to know whether indeed the LHb itself was exclusively hit across mice. So it is unclear where those fluorescently targeted LHb neurons project to (e.g. in LHb or in a perhaps surrounding structure also hit by the virus). Indeed as the authors also remark, it was surprising to find the PVT as an input. It may indeed be a real input, but it could also be cells picked up due to viral spread beyond LHb.

Response: We completely agree with the reviewer regarding the necessity of anatomical precision. This concern is precisely why we transitioned to using CRH-Cre::Ai14/Ai6 reporter mice in our subsequent experiments; this ensured that only CRF-expressing neurons within the LHb boundaries were manipulated or traced.

To address the reviewer's specific concern regarding the injection sites in Figure 1, we have now provided both low-magnification (zoomed-out) and high-magnification images

to demonstrate the high selectivity of our targeting. These images verify that our viral expression was restricted to the LHb also confirmed across all experimental cohorts. We utilize significantly smaller injection volumes compared to many standard protocols to ensure the viral spread is confined to our region of interest, as evidenced by the new representative images throughout the revised manuscript.

We have re-examined the retrograde labeling and confirmed the specific uptake within the LHb boundaries rather than viral spread to adjacent midline structures. Of note, a recent publication has identified the paraventricular nucleus of the thalamus (PVT) as a source of limbic input to the LHb[24], a projection that had not yet been characterized at the time of our initial submission. Our retrograde mapping findings are consistent with this new discovery, positioning the PVT-LHb pathway as a major component of the CRF inputs. Among the newly identified brain regions, we found no existing literature documenting a direct connection between the pretectal area (PRT) and the LHb, suggesting this may be a previously uncharacterized pathway. We have included this finding in our discussion, while also acknowledging that our retrograde strategy likely provides a conservative or "limited view" of the total CRF inputs. Specifically, certain pathways may be less amenable to retrograde transduction via rgAAV vectors. These clarifications have been integrated into the revised Discussion section to provide a more appropriate interpretation of our anatomical mapping.

ii. The other line of evidence is anterograde (e.g. Figure 3 and 4). Here the LHb is targeted, in CRF-cre mice, with a cre-dependent anterograde virus. The authors observe fluorescence, including likely of neurites, in LHb. Though here it's unknown if these contain synaptic endpoints so it is again unclear if they represent release sites. Also here PVT shows some fluorescence, but it is also unclear here whether this is an anterograde release site, or for instance cue to fluorescent cell bodies of PVTCRF neurons, targeted due to viral spread to such a nearby region.
To support the claim that LHbCRF neurons project within the LHb, additional evidence would be required. For instance, functional connectivity within the LHb, and/or forms of anatomical tracing that also show convergence of labeled neurites with markers for release sites.

Response: We thank the reviewer for this very valuable and constructive criticism regarding the distinction between fibers of passage and functional release sites. To definitively address whether the observed fluorescence represents functional local connectivity, we performed new optogenetic experiments (Figure 10) targeting the intra-LHb circuit. By expressing ChrimsonR specifically in LHb^{CRF} neurons and performing whole-cell patch-clamp recordings from neighboring non-CRF neurons, we observed robust, light-evoked excitatory postsynaptic currents (oEPSCs). This finding provided functional evidence that LHb^{CRF} neurons form local, excitatory synaptic connections

within the LHb. This functional confirmation bypasses the ambiguity of purely anatomical fluorescence and confirms that the neurites observed in our anterograde tracing are indeed functional release sites. Regarding the potential for viral spread into the PVT, our high-resolution AxioScan imaging and the use of the Ai6/Ai14 reporter lines confirmed that viral expression was strictly confined to the LHb boundaries. We observed a complete absence of labeled somata within the PVT in our anterograde tracing studies. Furthermore, we used the known projection targets of the PVT as an internal "leakage" control. The NAc and CeA are major projection targets of PVT neurons. Had our anterograde virus contaminated the PVT cell bodies, we would expect to see robust fluorescent axonal labeling and terminal fields within the NAc and CeA. The fact that these regions remained entirely clear of labeled fibers provides a high degree of confidence that our viral uptake was restricted to the LHb and was not the result of non-specific spread into the midline thalamus.

2. Further characterization of location of LHbCRF neurons.
The existence of LHbCRF neurons is interesting, and the field would benefit from a better understanding of where these cells exactly are in LHb and how numerous they are. Regarding position: Some figures suggest a rather medial clustering within the LHb, though other figures do not necessarily seem to report the same. I was surprised to find little if any reflection on this potentially interesting point. The paper would benefit from a dedicated analysis of the position within the LHb of the LHbCRF cells.

Response: We completely agree that a precise topographical map of this population is essential for understanding its circuit function. To address this, we performed a dedicated, large-scale RNAscope analysis across the entire rostrocaudal axis (5 AP levels).

Topographical Distribution and Position

Our new data (Figure 2-4 and Supplemental Figures 3–7) clarify that LHb^{CRF} neurons are primarily concentrated in the rostral portion of the LHb (Bregma -1.06 to -1.34 mm). Within these levels, the population exhibits a distinct spatial organization: a dense cluster is situated in the medial LHb (LHbM), bordering the medial habenula, while a significant subpopulation extends into the lateral LHb (LHbL). This dual medial-lateral presence at rostral levels likely explains the perceived discrepancies in earlier figures that focused on single planes. As the axis moves caudally (Bregma -1.58 mm), the population density decreases significantly.

Unbiased Quantification and Sex Differences

To ensure our quantification was not confounded by potential structural dimorphism, we analyzed the total surface area of the LHb across all 5 AP levels using the Allen Brain Atlas and Paxinos templates. We found no significant differences in LHb surface area between sexes (Supplementary Figure 3), allowing for a truly unbiased comparison of

cell counts. Our results show that while the topographical distribution is identical between sexes, male mice possess a significantly higher absolute number of LHb^{CRF} neurons than females (Figure 4).

Neurochemical and Functional Validation

We further characterized these cells by their transcriptional profile, confirming that 100% express vGlut2. Approximately 17–18% co-express GAD2, while a very small minority (primarily in males) co-express vGlut2 and vGAT. Crucially, our optogenetic data (Figure 10) provides functional evidence for this topography; we recorded light-evoked excitatory currents from local non-CRF neurons, confirming that these rostral LHb^{CRF} cells form a functional, excitatory intra-habenular circuit, with females exhibiting significantly stronger synaptic connections. We have updated the manuscript to include these dedicated positional analyses and believe this comprehensive mapping significantly strengthens the anatomical foundation of our study.

Regarding number: It remains a bit unclear how big or small this population is. In some figures (e.g. Figure 2 and 3) the amount of LHbCRF cells seems to be very low. Though again, in other figures this looks a bit different. Figure 2 is also missing quantification in general. There are statements in the text about this Figure, such as that 100% of neurons are vglut2+ and that "only a small minority" has VGAT, but there is no information on the numbers behind these results as far as I can tell. What was the number of mice used here? The number of cells evaluated, with which number/percentage VGAT positive, etc?
These quantifications of position and number should ideally be done separately for the RNA scope data and for an experiment where those cells were instead virally captured, thereby also giving some insight in whether those two independent ways of showing the cells indeed converge on (within reason) a similar result and on the same population.

Response: We thank the reviewer for this outstanding and constructive suggestion. As mentioned in our response to your previous comment, we have taken this feedback to heart and performed a new, comprehensive RNAscope study in transgenic CRH-Cre::Ai14 mice, which enabled us to precisely quantify the number, location, and neurochemical identity of these neurons across both sexes. Our quantification (Figure 4) confirmed that LHb^{CRF} neurons are a discrete but consistent population (n=13 mice), with 100% co-expression of VGlut2 and a negligible minority (~<3%) expressing vGat. By analyzing 5 AP levels, we found that the perceived low numbers in earlier figures were due to the significant concentration of these cells in the rostral LHb (Bregma -1.06 to -1.34), where they are most numerous. Furthermore, the cell counts obtained via RNAscope converge closely with the numbers "captured" via our Ai14/Ai6 reporter lines in the DREADD and optogenetic experiments, providing confidence that both independent methods target the same excitatory population. We have replaced Figure 2

in earlier submission with Figure 2-4 and Supplemental Figures 3–7 and the Results section to include these specific cell counts and percentages.

3. In general the microscopy images for histology can be improved in some regards.
Somewhat linked to point 2 above, in many figures the paper would benefit from seeing more of the LHb than currently the case. In Figure 1 we do not see the lateral extremities of the LHb, making it hard to judge if there are retrolabeled cells there or not. In figures 2 and 3 instead we see but a small fragment of the LHb, missing most of the ventral part. Figure 5 suddenly opts for sagittal representation of the LHb. Not inherently a problem, but of course making it difficult to see where along the medial-lateral axis we are, which may be, as described above, a very relevant factor. Figure 6 images could benefit from labelling of regions for easier understanding of positioning.

Response: We sincerely apologize for the initial image quality and the lack of low-magnification overviews, which obscured the anatomical context of our findings. We have completely revamped our histological presentation to provide the clarity and regional labeling requested. To address these concerns, we transitioned from standard fluorescence microscopy to high-resolution Zeiss AxioScan imaging. This allowed us to generate large-scale, mosaic images of the entire LHb in every section, ensuring that the medial, lateral, and ventral boundaries are fully visible. Specifically, we have implemented the following improvements: Expanded Field of View: All figures now feature "zoomed-out" overviews of the entire LHb. This allows for a clear assessment of LHb^{CRF} distribution across the lateral and medial extremities and ensures that no retro-labeled or fluorescent cells are omitted from view. We have overlaid brain atlas borders and included Bregma levels on all representative images. This provides a precise spatial coordinate system for the reviewer to judge cell locations along the rostrocaudal and mediolateral axes. We have optimized the fluorescence intensity and adjusted the gain to provide brighter, clearer signals while maintaining low background levels, ensuring that even sparse cell populations are accurately represented. In all figures throughout our targeting validations, we have prioritized coronal sections to maintain consistency in our topographical reporting and included corresponding coronal schematics to clarify the medial-lateral positioning. We are grateful for these valid points, as the enhanced resolution and anatomical labeling provided by the AxioScan system have significantly improved the rigor and interpretability of our spatial mapping data.

4. Validity of statistical analysis approach.
The authors use relatively low N experiments in many of the figures. For instance a lot of the tracing studies are done with n=3, and then it is also not entirely clear how the variability between those n=3 was. For instance in Figure 1, though it also applies to other figures, it's not clear if this distribution of found percentages

was consistent across animals or not. This should be clarified.
Most importantly, for the main behavioral test where an effect is found (action locking / freezing) some questions arise about the statistical approach taken. It is not entirely clear to me whether here the N used represents trial number, or animal. It would seem to be trial number. In my view animal should be the independent unit on which the statistical analysis should be run for this experiment. Whether this is the case would need to be clarified, and if it is not done on animal then it would likely need to be reanalyzed in that way.

Response: We thank the reviewer for these critical points regarding statistical transparency and rigor. We have addressed these concerns by re-evaluating our datasets and updating our analysis to prioritize the individual animal as the independent experimental unit.

1. Retrograde Tracing and Variability (Figure 1): We have re-analyzed the retrograde tracing data using a combination of the Allen Brain Atlas and Paxinos templates for more precise regional quantification. To address the question of consistency, we have updated Figure 1 and its associated supplementary data to show the individual variability across our cohort (n=3; 2 males, 1 female). While the small N reflects the breeding challenges previously discussed, the updated figures now use unique symbols for each animal, allowing for a transparent assessment of how consistent the projection percentages were across the individual subjects.

2. Behavioral Analysis (VLST): Regarding the Visual Looming Shadow Test (VLST), we appreciate the reviewer's guidance on the appropriate unit of analysis. Of note, based on R1's and your recommendation, we re-evaluated our Visual Looming Shadow Test (VLST) videos and identified three distinct behavioral outcomes: (1) escape, (2) action-locking, and (3) action-locking that transitions to escape in which mice exhibit an immediate freezing behavior followed by a delayed escape to the shelter. Our previous analysis had grouped the third category within general "escapes." By separating these, we confirmed our intimal observation that LHb^{CRF} activation significantly shifts the defensive profile toward action-locking in both sexes. Specifically, our new analysis (now presented in Figure 8) demonstrates: Chemogenetic activation significantly increased the frequency of trials resulting in action-locking across both males and females. LHb^{CRF} activation prolonged the duration of action-locking behaviors in both sexes, regardless of whether they eventually transitioned to an escape. We added an analysis of shelter stay duration as a measure of post-escape safety-seeking. We found that activation in females specifically resulted in a protracted shelter stay, suggesting heightened threat perception or delayed recovery compared to males[9]. For statistics, data for latencies and action-locking duration and shelter stay: We have moved away from trial-based pooling for our primary statistical comparisons. We now calculate the mean per animal for escape latency, shelter stay duration, and action-locking duration. These per-animal averages were then analyzed using a two-way ANOVA (Sex x

Treatment). This shift to animal-based N not only aligns with the reviewer's suggestion but actually revealed more subtle effects that have strengthened our original findings. Categorical Data (Action-Locking vs. Escape vs. Action-locking to Escape): For the Chi-square analysis and associated pie charts, we have retained the trial-based approach, as this is the standard statistical convention for categorical transitions in the looming literature [8-11] to capture the stochastic nature of defense selection. Clarification on Sample Sizes: We have also added a clarifying note in the figure legends: three mice (one male Gq+JHU, one female Gq+Saline, and one female Gq+JHU) did not exhibit any action-locking behavior across all trials. Consequently, the N for the "Action-Locking Duration" analysis is n-1 for those specific groups, as only mice that exhibited the behavior could be included in the duration calculation.

3. Transcriptional Quantification (RNAscope): For the new RNAscope study, we have ensured high statistical power by including 7 male and 6 female CRH-Cre::Ai14 mice. All neurochemical percentages and cell counts are reported as the mean of these independent biological replicates, providing a robust foundation for our claims of sexual dimorphism in LHb^{CRF} population size.

We believe these updates significantly enhance the statistical transparency of the manuscript and ensure that our behavioral conclusions are based on rigorous, animal-independent units of measure.

5. Female vs Male
I appreciate that the authors address potential differences between females and males in the behavioral tests. What is aside from that rather striking however is that Figure 3 (males) vs Figure 4 (females) seem to yield a very different situations for LHbCRF neurons, at least in the representative images shown. It is unclear whether (if this optical difference is indeed representative) this is due to differences in virus (AAV8-DIO-mCherry vs AAV5-EF1a-ChR2-EYFP) or in a difference between males and females. The authors need to clarify whether between males and females the LHbCRF populations are similar or different in their size/position. This to complement their functional analysis in Figure 8.

Response: We thank the reviewer for this critical observation. The perceived differences in our initial Figures 3 and 4 were indeed confounded by the use of different viral constructs and fluorophores, which made a direct anatomical comparison between sexes difficult. To rectify this, we have moved to a standardized approach for all our anatomical and functional mapping in this revision.

1. Anatomical Characterization (RNAscope)

To provide a definitive answer regarding the size and position of the LHb^{CRF} population, we conducted a comprehensive RNAscope study in 13 mice (7 males, 6 females). This method allowed us to visualize the endogenous transcript without the variability of viral tropism or expression levels. As shown in our new Figure 4, we found that while the

topographical distribution (primarily rostral and medial) is identical between sexes, there is a significant biological difference in population size: males possess a higher absolute number of LHb^{CRF} neurons than females.

2. Standardized Anterograde Mapping

In our revised anterograde tracing experiments, we transitioned to a uniform strategy using AAV5-Syn-FLEX-ChrimsonR-tdTomato in CRH-Cre::Ai6 mice of both sexes. This uniform approach not only confirmed the primary projection targets identified in our initial experiments (e.g., RMTg, SNc, and PAG) but also enabled the identification of additional downstream regions that were previously underrepresented, including the Superior Colliculus (SC), Ventral Tegmental Area (VTA), Median Raphe (MnR), and Dorsal Raphe (DR). Crucially, these projection patterns were found to be consistent between males and females. We have updated the manuscript with high-resolution, zoomed-out AxioScan images for both sexes, providing a transparent and directly comparable view of these target areas. By using the same viral construct and high-sensitivity reporter lines across all cohorts, we have eliminated the confounding variables associated with our previous mixed-viral strategy, ensuring that our anatomical characterization is both robust and reproducible.

3. Functional Divergence

While the topographical organization of projections is similar, our optogenetic data (Figure 10) revealed that the *synaptic strength* and *local connectivity* are sexually dimorphic. Specifically, female LHb^{CRF} neurons form stronger local excitatory connections than their male counterparts, providing possible physiological basis for the observed behavioral phenotypes in the VLST.

We have revised the Results and Discussion to explicitly clarify that while the anatomical organization of these neurons is conserved across sexes, significant differences exist in total cell number and synaptic efficacy.

6. Are there reported effects of stimulation of LHbCRF neurons mediated by CRF? Finally, it remains unclear whether any of the observed or inferred effects of LHbCRF neurons (e.g. local or distal connectivity, action locking) involve any CRF signaling. Since these are vglut2+ neurons that are also positive for CRF, a potentially dual mode of action is possible for these cells. There are currently no hints as to what the contribution of CRF is. An inclusion of an experiment addressing whether CRF is actually released and relevant for the role of these neurons, would be a strong added value to the work.

Response: This is an outstanding and forward-looking question. We agree that the potential for glutamate/CRF corelease, involving both fast glutamatergic transmission and slower peptidergic neuromodulation, is a compelling characteristic of CRF+ circuits. Due to the significant constraints on our animal colonies following the breeding

disruptions mentioned previously, we were unable to perform the complex, high-resource experiments required to definitively isolate CRF peptide signaling. A rigorous assessment would necessitate high-frequency optogenetic stimulation protocols, the use of floxed-CRH mice, or the implementation of CRF GRAB sensors to detect real-time peptide release. While we find this question of utmost importance, we believe it is currently beyond the scope of the present study. However, we have provided a substantial amount of new functional and anatomical data that significantly advances the field's understanding of this population. We hope the reviewer appreciates that the depth of the new physiological, anatomical, and behavioral data provided in this revision provides a robust foundation for the role of LHb^{CRF} neurons. We fully intend to investigate the specific contribution of peptidergic signaling in our future studies.

Minor point

1. Line 126 – 128: The sentence: "Notably, retrograde labeling was not detected in some other known CRF-expressing regions that project to the LHb, such as the VTA and CeA, supporting the rgAAV tracer's specificity" (similar argument in L267 and beyond) I find confusing.

In my view this should not be cited as evidence for specificity of the tracer. It's not an actual negative control, because we don't know if those CRF+ VTA and CeA cells indeed shouldn't project to LHb. In the meantime we know that retro AAV generally has considerable problems of tropism, labelling certain pathways much better than others. So it is very possible that these inputs do actually exist, but they are just not targetable via rg-AAV. That could also be viewed as a form of specificity, but probably not in the typical sense of the word, which is that the strategy would be specific if it doesn't have any off-target labelling (of non-CRF neurons). These sentences should therefore be amended. Moreover, the discussion should more clearly discuss the potential limitation of viral tropism giving a limited view on potential CRF inputs to LHb (in Fig 1) due to the sole use of retroAAV for retrograde mapping there.

Response: We thank the reviewer for this insightful correction. We agree that the absence of labeling in the VTA and CeA does not constitute a formal negative control for specificity, as the lack of signal could indeed be a result of the differential tropism or stochastic labeling inherent to retrograde AAVs. It is entirely possible that these inputs exist but were not captured by this specific viral serotype. We have amended the manuscript to remove the claim that these findings support the "specificity" of the tracer. Furthermore, we have updated the Discussion to explicitly address the limitations of relying solely on retrograde AAV for circuit mapping. Of note, a recent publication has identified the paraventricular nucleus of the thalamus (PVT) as a source of limbic input to the LHb[24], a projection that had not yet been characterized at the time of our initial submission. Our retrograde mapping findings are consistent with this new discovery,

positioning the PVT-LHb pathway as a major component of the CRF inputs. Nevertheless, we now acknowledge that our data likely provides a conservative or "limited view" of the total CRF inputs to the LHb, as certain pathways may be less amenable to retrograde transduction via this strategy. We believe these changes more accurately reflect the technical boundaries of current viral tracing methods and appreciate the reviewer's guidance on this point.

References

1 Lin, J. Y., Knutsen, P. M., Muller, A., Kleinfeld, D. & Tsien, R. Y. ReaChR: a red-shifted variant of channelrhodopsin enables deep transcranial optogenetic excitation. *Nature neuroscience* **16**, 1499–1508 (2013). https://doi.org/10.1038/nn.3502

2 Goyer, D. & Roberts, M. T. Long-range Channelrhodopsin-assisted Circuit Mapping of Inferior Colliculus Neurons with Blue and Red-shifted Channelrhodopsins. *J Vis Exp* (2020). https://doi.org/10.3791/60760

3 Mathis, V. P., Williams, M., Fillinger, C. & Kenny, P. J. Networks of habenula-projecting cortical neurons regulate cocaine seeking. *Science Advances* **7**, eabj2225 (2021). https://doi.org/doi:10.1126/sciadv.abj2225

4 Simmons, S. C. *et al.* AKAP150-anchored PKA regulates synaptic transmission and plasticity, neuronal excitability and CRF neuromodulation in the mouse lateral habenula. *Communications Biology* **7**, 345 (2024). https://doi.org/10.1038/s42003-024-06041-8

5 Flerlage, W. J. *et al.* Effects of Repetitive Mild Traumatic Brain Injury on Corticotropin-Releasing Factor Modulation of Lateral Habenula Excitability and Motivated Behavior. *Journal of Neurotrauma* (2024). https://doi.org/10.1089/neu.2024.0184

6 Authement, M. E. *et al.* A role for corticotropin-releasing factor signaling in the lateral habenula and its modulation by early-life stress. *Science signaling* **11** (2018). https://doi.org/10.1126/scisignal.aan6480

7 Levinstein, M. R. *et al.* PACAP-expressing neurons in the lateral habenula diminish negative emotional valence. *Genes Brain Behav*, e12801 (2022). https://doi.org/10.1111/gbb.12801

8 Sandoval, K. C., Rychlik, J. & Choe, K. Y. Calcium Dynamics in Hypothalamic Paraventricular Oxytocin Neurons and Astrocytes Associated with Social and Stress Stimuli. *eNeuro* **12** (2025). https://doi.org/10.1523/ENEURO.0196-24.2025

9 Daviu, N. *et al.* Paraventricular nucleus CRH neurons encode stress controllability and regulate defensive behavior selection. *Nature neuroscience* **23**, 398–410 (2020). https://doi.org/10.1038/s41593-020-0591-0

10 Lecca, S. *et al.* Heterogeneous Habenular Neuronal Ensembles during Selection of Defensive Behaviors. *Cell reports* **31**, 107752 (2020). https://doi.org/10.1016/j.celrep.2020.107752

11 Rasiah, N. *et al.* Homeostatic scaling ensures behavioural stability during corticosterone negative feedback. *Molecular psychiatry* (2025). https://doi.org/10.1038/s41380-025-03391-5

12 Madisen, L. *et al.* A robust and high-throughput Cre reporting and characterization system for the whole mouse brain. *Nature neuroscience* **13**, 133–140 (2010). https://doi.org/10.1038/nn.2467

13 Madisen, L. *et al.* Transgenic mice for intersectional targeting of neural sensors and effectors with high specificity and performance. *Neuron* **85**, 942–958 (2015). https://doi.org/10.1016/j.neuron.2015.02.022
14 Chen, Y., Molet, J., Gunn, B. G., Ressler, K. & Baram, T. Z. Diversity of Reporter Expression Patterns in Transgenic Mouse Lines Targeting Corticotropin-Releasing Hormone-Expressing Neurons. *Endocrinology* **156**, 4769–4780 (2015). https://doi.org/10.1210/en.2015-1673
15 Füzesi, T., Daviu, N., Wamsteeker Cusulin, J. I., Bonin, R. P. & Bains, J. S. Hypothalamic CRH neurons orchestrate complex behaviours after stress. *Nature communications* **7**, 11937 (2016). https://doi.org/10.1038/ncomms11937
16 Birnie, M. T. *et al.* Stress-induced plasticity of a CRH/GABA projection disrupts reward behaviors in mice. *Nature communications* **14**, 1088 (2023). https://doi.org/10.1038/s41467-023-36780-x
17 Paretkar, T. & Dimitrov, E. The Central Amygdala Corticotropin-releasing hormone (CRH) Neurons Modulation of Anxiety-like Behavior and Hippocampus-dependent Memory in Mice. *Neuroscience* **390**, 187–197 (2018). https://doi.org/10.1016/j.neuroscience.2018.08.019
18 Birnie, M. T. *et al.* Stress-induced plasticity of a CRH/GABA projection disrupts reward behaviors in mice. *Nature communications* **14**, 1088 (2023). https://doi.org/10.1038/s41467-023-36780-x
19 Van Savage, J. & Avegno, E. M. High dose administration of DREADD agonist JHU37160 produces increases in anxiety-like behavior in male rats. *Behavioural brain research* **452**, 114553 (2023). https://doi.org/10.1016/j.bbr.2023.114553
20 Flerlage, W. J. *et al.* Involvement of Lateral Habenula Dysfunction in Repetitive Mild Traumatic Brain Injury-Induced Motivational Deficits. *J Neurotrauma* **40**, 125–140 (2023). https://doi.org/10.1089/neu.2022.0224
21 Bonaventura, J. *et al.* High-potency ligands for DREADD imaging and activation in rodents and monkeys. *Nature communications* **10**, 4627 (2019). https://doi.org/10.1038/s41467-019-12236-z
22 Flanigan, M. E. *et al.* Orexin signaling in GABAergic lateral habenula neurons modulates aggressive behavior in male mice. *Nature neuroscience* **23**, 638–650 (2020). https://doi.org/10.1038/s41593-020-0617-7
23 Quina, L. A., Walker, A., Morton, G., Han, V. & Turner, E. E. GAD2 Expression Defines a Class of Excitatory Lateral Habenula Neurons in Mice that Project to the Raphe and Pontine Tegmentum. *eNeuro* **7** (2020). https://doi.org/10.1523/eneuro.0527-19.2020
24 Cui, J., Qian, J., Wang, J. & Shi, L. Decoding the dual regulatory systems: Contemporary insights into the lateral habenula and rostromedial tegmental nucleus physiology. *Neurobiology of Disease* **214**, 107049 (2025). https://doi.org/https://doi.org/10.1016/j.nbd.2025.107049
