# Supplementary material for: Neuroanatomical and behavioral characterization of corticotropin-releasing factor-expressing lateral Habenula neurons in mice
Source: Commun Biol. 2026 Jun 16;9:1280. doi: 10.1038/s42003-026-10490-8 (PMC13631236; doi:10.1038/s42003-026-10490-8)
Supplement: Supplementary file 3 [file 42003_2026_10490_MOESM3_ESM.pdf]

Description of Additional Supplementary File

File name: Supplementary Data

Description: Supplementary data file includes dataset related to Figures 1-10.
